# Insights from homeless men about PRISM, an innovative shelter-based mental health service

**DOI:** 10.1371/journal.pone.0250341

**Published:** 2021-04-22

**Authors:** Brigitte Voisard, Rob Whitley, Eric Latimer, Karl Looper, Vincent Laliberté

**Affiliations:** 1 Department of Psychology, Université du Québec à Montréal, Montreal, Quebec, Canada; 2 Department of Psychiatry, Douglas Mental Health University Institute, McGill University, Montreal, Quebec, Canada; 3 Lady Davis Institute for Medical Research, Jewish General Hospital, Montreal, Quebec, Canada; 4 Department of Psychiatry, Faculty of Medicine, McGill University, Montreal, Quebec, Canada; University of Birmingham, UNITED KINGDOM

## Abstract

PRISM (*Projet Réaffiliation Itinérance Santé Mentale*–mental health and homelessness reaffiliation project), is a new shelter-based mental health service in Montreal, Canada. It offers short-term residential services in a shelter with the aim of housing and connecting the person to the appropriate services in the community. This qualitative research project was designed to gain a rich understanding of service-user experience within this program, and to apply these impressions to a broader reflection concerning how to best serve the needs of homeless people living with severe mental illness. We conducted in-depth interviews with 20 clients from the all-male PRISM-Welcome Hall Mission at program intake and departure between May 2018 and March 2019. We used methods stemming from grounded theory to analyze themes emerging from the interviews. Analysis revealed three core aspects endorsed by PRISM clients as helpful to their recovery: first, the community-based and flexible PRISM structure allows for continuity in daily routine through the preservation and expansion of the client’s existing informal resource network; second, the secure environment is conducive to improving one’s physical and mental health; and third, the multimodal mental health and social service approach used at PRISM is appreciated and stands in contrast to what most have experienced during other inpatient experiences. This led us to reflect more broadly on the benefits of a shelter-based intervention, as a catalyst to the achievement of longer-term goals such as housing, as well as flexible care adapted to the specific needs of these individuals. Even though this study took place in a specific program in Quebec, it sheds light more broadly on how to best meet the needs of individuals with mental illness living in homeless situations and contributes to the growing literature on men’s mental health.

## Introduction

The term severe mental illness (SMI) refers to mental disorders that share characteristics of strong symptom severity and significant functional impairment when untreated [1]. People living with SMI are overrepresented in the homeless population [2, 3]. Mental health services within the Canadian public health system have long been poorly equipped to answer the needs of this population [4–6]. Even though symptoms of mental illness may be exacerbated by the harshness of life on the street, homeless individuals may ignore their own mental health needs in favour of more pressing survival concerns [7]. Barriers to treatment for homeless individuals with mental illness include difficulty keeping up with medical appointments or communicating with the treatment team, distrust of psychiatric or social services, as well as the multiplication of unforeseen circumstances that may occur in a homeless situation [8–10]. Furthermore, individuals with mental illness who are homeless at hospital discharge have a significantly higher risk of readmission, suggesting that interventions that promote an efficient transition to outpatient care are especially important in this population [11].

Recent decades have seen a shift in terms of best practices both in the way we tackle homelessness and in our approach to mental healthcare. First, strategies concerning homelessness have moved away from treatment first (TF) frameworks and towards approaches that favour community housing accompanied by a supportive network. This includes the Housing First model as well at the Critical Time Intervention (CTI) model. The former aims to provide individuals with stable housing as quickly as possible while connecting them to necessary services [12, 13]. The latter is geared towards providing support to individuals as they transition from institutions (hospitals, shelters, etc.) towards the community, as they are “called upon to navigate a complex and fragmented system of care” [14, p. 297]. Second, the recovery model for mental illness has gained increasing acceptance. It includes a focus on creating a patient-driven treatment plan to enable people with mental illness to live a fulfilling life in the community [15, 16]. As such, individuals must be empowered to take an active part in a treatment that is not defined by the treatment of symptoms but more broadly by the accomplishment of objectives that are meaningful to the client, on the basis of their own personal and environmental strengths [17].

The *Projet de Réaffiliation en Itinérance et Santé Mentale* (PRISM) (mental health and homelessness reaffiliation project) is an innovative service launched in Montreal, Canada in 2013. PRISM is designed to serve homeless individuals with severe mental illnesses such as schizophrenia, schizoaffective disorder, major depression with psychotic symptoms and bipolar disorder. Comorbidities, notably with substance use, are also common. PRISM is inspired by the Housing First and CTI models. The program operates at three sites in Montreal, Canada, offering a total of 10 beds for women and 26 beds for men. The PRISM—Welcome Hall Mission (WHM), the site of the present study, is a partnership between the shelter and the CIUSSS West-Central, a health district that includes the Jewish General Hospital. It offers 8 beds for men in an augmented shelter accommodation (assigned single beds and lockers, increased privacy, lounge area, small dormitory).

Compared to most other forms of inpatient treatment or shelter accommodation, life at PRISM is not strictly regulated: before the 11PM curfew, clients are free to come and go as they please. While cigarettes are allowed outside the building, drugs and alcohol are not permitted anywhere on the premises, and clients are expected not to appear overly impaired by substance use on-site. The daily schedule is not regimented apart from meal hours (which clients are free to attend or not) and meetings with staff. Intake occurs mostly through outreach within the adjoining emergency shelter and through referrals by partner hospitals. The on-site approach to care is focused toward recovery: a part-time psychiatrist, a part-time nurse, a full-time psychoeducator or social worker and a full-time shelter case worker support each individual through the ascertainment of their goals, the provision of mental healthcare and the aid needed to move on to the most appropriate available services. The psychiatrist at PRISM-WHM meets with clients one or twice a week. He uses an eclectic therapeutic array, adapted to the needs, challenges and strength of each client. In most cases, this involves psychopharmacology and supportive counselling. When possible, the psychiatrist also conducts psychotherapy that focuses on interpersonal relationships, defining new goals, trauma, or addiction, without adhering to a strict psychotherapeutic format. Through the weekly sessions, he works at providing a *holding environment* in which suffering has meaning [18]. The nurse manages on-site health evaluations, blood tests and medication, and acts as liaison with other medical teams. The psychoeducator helps clients find housing, connect with other services, and prepare to adapt to new circumstances. As a trained mental health professional, she also participates in counselling related to the daily challenges that clients may experience throughout the various steps of the reaffiliation process. Together with the case worker, they provide emotional support and guidance through a full-time presence on-site. The case worker also acts as a liaison with the other shelter workers.

This qualitative study aims to gain insights from users of the PRISM in Montreal into their perception of the program in relation to their previous experience of mental health and homelessness resources. These impressions are applied to a broader reflection concerning how to best serve the needs of homeless people living with SMI.

## Methods

Qualitative methods have been identified as ideally suited for research involving vulnerable populations, as they foreground the voices of participants and allow for in-depth accounts of their experiences [19, 20]. Rich insights from homeless individuals have led to a deeper understanding of homelessness, its causes and the quality of services offered [21–23]. Services designed in isolation from the insights of their principal stakeholders–as is often the case in the context of homelessness–are at risk of being disconnected from the needs of their clientele [21]. This study uses qualitative methods stemming from Glaser and Strauss’ grounded theory [24] and adapted by Paillé [25]. These methods aim to describe and analyze experiences in order to produce a theoretical framework that is grounded in qualitative data. As such, it assumes that there is an underlying logic to the studied subject that can emerge from the perceptions and attitudes of its actors. Uncovering this logic requires a gradual and iterative process whereby the emerging theory is constantly compared and validated with the observed reality [25]. Here, we present an in-depth analysis of emergent themes aiming to build an understanding of user experience within the PRISM model, and to then place these results in the context of homelessness literature to produce a theoretical framework for this type of model.

### Recruitment

Ethics approval for this study was obtained from the Psychosocial Research Ethics Committee of the CIUSSS West-Central Montreal Research Ethics Board (REB). From May 31, 2018, to March 22, 2019, each of the 30 new PRISM-WHM clients were invited to take part in this study. Clients were met by a member of the research team (BV) within two weeks of entering the program, at which point the aims of the project were explained and participation requirements were detailed. As participants were all impacted at different levels by mental illness, special care was given to going over the consent form with each participant in order to clarify language, answer questions and confirm their understanding. The only inclusion criterion was to be admitted to the PRISM-WHM, which has a capacity of 8 male participants. No participant had to be excluded due to insufficient mastery of French or English. Over these 10 months of data collection, only 2 new PRISM clients did not consent to the interviews. One person consented to the research program but left before the first interview. Seven participants left the program before a second interview was possible or had yet to complete an exit interview when we started data analysis. The testimonies of the remaining 20 participants who completed both intake and departure interviews were included in this analysis.

### Data collection

Once informed written consent had been given, participants could choose to complete an interview right away or schedule another meeting. The semi-structured interview at intake centred around their previous history, including questions such as (i) can you tell me about the first time you found yourself in a homeless situation? (ii) can you tell me about the services (social and mental health) you’ve received since you started experiencing housing instability? (iii) what have been your biggest obstacles, and on the contrary, what have you found to be helpful? Participants subsequently completed a sociodemographic questionnaire containing information about their age, educational level, sexual orientation, housing history, substance use history and criminal justice history. The exit interview mainly addressed their experience within PRISM, including questions such as (i) can you tell me generally if/what impact the program had on you? (ii) can you tell me about your experience at the PRISM? (iii) can you tell me if/how the program impacted your integration within society? On average, interviews lasted 30 to 45 minutes. All interviews were conducted by one author (BV), a graduate student in clinical psychology, who was trained and supervised by the other authors. All diagnoses were made by psychiatrist VL, and were gathered as part of the chart review.

### Data analysis

Each qualitative interview was led, recorded, and transcribed as soon as was possible by BV. Pseudonyms were attributed to each participant. Two members of the research team (BV and VL) met regularly throughout the data collection period to discuss emerging possible emerging topics. While interview questions for Times 1 and 2 were planned ahead as guidelines for the conversation, participants brought forth many insights that went beyond these initial queries. At the end of the data collection period, all transcripts were reread in order to create initial inductive codes. This initial coding scheme was agreed upon and tested on a sub-sample of interview transcript using MAXQDA 2018 [26], a computer assisted qualitative data analysis software. This sub-sample test enabled us to identify further sub-ordinate codes and create the final coding scheme. All transcripts were then coded by BV using the same software. We evaluated which codes were most prominent and fitting to our research objectives. Overlapping codes were then grouped into wider themes. Graphic representation was used as a brainstorming tool to explore how these themes were connected to PRISM and to more general realities of homelessness. These relationships were discussed between BV, VL, EL and RW to arrive at the three themes described in the results section. All analyses were conducted in the interview’s original language (English or French), and French quotes were translated into English for this article by the first author, who is fluently bilingual. All quotes are available in their original language in the S1 Annex.

As is standard procedure, the qualitative data is not quantified. As such, precise numbers of individuals who mentioned a theme are not given. A sense of the theme’s significance in the data is provided by use of descriptive language in the text indicating the prominence of the theme.

The demographic information was extracted both from the demographic portion of the qualitative interview as well as from a chart review. To protect confidentiality, demographic information pertaining to fewer than 5 participants is simply quantified as n<5.

## Results

### Demographic characteristics

Participants were men aged 19 to 65 at intake, with a mean age of 44.15. Years of education ranged from 7 to 16 with a mean of 11.55. The vast majority of participants identified as heterosexual, with a small minority (n<5) identifying as bisexual. Participants were not asked about gender identity, and no participant raised the issue during interviews. A majority of participants (13 out of 20) arrived at PRISM after a period living on the streets and/or using homeless shelters. The remainder were referred to the PRISM by a social worker after a hospital stay or, in a small minority of cases (n<5), an eviction. Among the 20 participants, most received a psychotic disorder as their main diagnosis while a minority (n<5) were diagnosed with bipolar affective disorder type 1. All participants already had a psychiatric history of SMI prior to being admitted at PRISM. Furthermore, all had a history of psychiatric hospitalization. Almost all participants were already receiving some form of social assistance revenue at intake (n>15). A majority reported having experienced substance abuse in their lifetime (12), 5 in the past 6 months. Only 5 out of 20 did not report any experience with law enforcement in their lifetime (including tickets, misdemeanours, criminal offences or prison time).

### Core findings

Our analyses revealed that participant impressions of the short-term intervention provided at PRISM had to be understood through the lens of their prior experiences with housing instability as well as with hospitals. As such, alluding to these various experiences on the street and in psychiatric hospitals was necessary in order to make sense of participants’ experiences and perceptions of PRISM. Three major themes emerged from our analyses: the importance of accommodating informal resource networks, of offering a “break” from chasing after basic needs of food and shelter, and of a multimodal form of care that could answer a variety of needs.

#### Accommodating informal networks

The first emergent theme was that of a certain continuity between the myriad resources and personal strategies used to survive on the street or through housing instability and those developed during time spent at PRISM. Indeed, our analyses suggest that PRISM was not experienced as a categorical rupture from previous routine, allowing instead for a continued investment in some aspects of the informal networks built by each individual. As participants transitioned towards stable housing, their testimonies highlighted the importance of the balance achieved by PRISM between the maintenance of some of these personal patterns and a simplified access to formal resources as participants.

When asked about the kinds of resources used prior to entering the PRISM program, it became clear that participants were not thinking only in terms of the “formal offer of services” available in the city. An overwhelming majority of our participants described crafting a custom assemblage out of both formal and informal resources. André (38) used a video game analogy to illustrate how he made decisions about navigating the streets. (All participant names have been changed.)

“It’s like is I was playing Zelda or Link, right? So, I have a frame, you know, I can go click the frame and go for a walk […], I can go to the store, at the store there’s a computer, I’ll go sit in my corner, go to another corner, get down, go get my coffee, because I went—I went to the other store, I didn’t get my coffee there, because I don’t get a refill.” (André, 38 translated from French)

As such, other than shelters and community-based programs, places to stay for the night or rest during the day could include inexpensive hotels, parks, libraries and university campuses. Almost all participants identified fast-food restaurant as helpful resources, especially when they allowed one to rest for a few hours unbothered or even tolerated taking water or juice:

“It was so helpful, for real, I never drank so much juice in my life! I was there, I would take some juice, no one said anything. […] It might sound stupid, but they would accommodate me, like, I knew a few McDonalds that never kicked me out”. (Frédéric, 26, translated from French.)

Similarly, while many participants were receiving government financial aid at intake, they could also rely on friends or family, panhandling, returning empty bottles and cans, and sometimes stealing. As such, each participant built his network based on what was useful and clearly available.

When talking more specifically about their stay at PRISM, testimonies revealed that many participants preserved aspects of these informal networks. Frédéric (26) and Chi (55), for example, found housing through acquaintances, not through the counsellor on-site. Furthermore, many participants maintained routine habits: “We pick up bottles together, and… Well, yesterday we made five bucks each. Five bucks is five bucks” (Rémi, 65, translated from French). Outings for food or coffee at a fast-food restaurant also remained frequent.

Furthermore, cooperation between clients was present and took different forms including trading objects, running errands in exchange for money, or even the occasional gift:

“[other participant], he’s the one who gave me the shoes… and, euh… I gave him cigarettes often, you know… I had some money and I gave him cigarettes, so he gave me shoes and like… a shirt and everything. So we were kind of like friends.” (Frédéric, 26, translated from French.)

As such, group life could facilitate the preservation of informal networks. Even more importantly, this communal experience could enable participants to expand these networks through new interpersonal bonds: “I had a negative experience [with another client] but it became positive. And that, I’m proud of that. We understood each other. And now, he’s leaving this morning, and I have a heavy heart.” (Marcel, 64, translated from French.)

“Breaking my isolation, that’s what I had to do. Breaking my isolation and making new friends, and… Since we’re in a group–there are eight of us, right–well, we don’t have a choice but to coexist, you know, we have to talk and everything.” (Stéphane, 47, translated from French.)

Participants spoke appreciatively of the program’s downtown location, which also facilitated the maintenance of routine habits:

“I met new friends. Uhm… I like the food here, I like the food here, yeah, the food is good. Uhm… it’s downtown, you know, it’s near the metro, it’s easy to get around from here. Yeah, there’s… there’s a lot of good things about it, yeah.” (Marc, 51, original English quote)

For Rémi, this influenced his decision to stick with the program: “Well, now, I don’t want to leave as much, because I’m eating very well, here I can take the metro, to go to the post office [P.O. box] every day, as it is now I’m going three times a week” (Rémi, 65, translated from French).

Another participant spoke about expanding his activity network by participating in shelter life on-site: “Sometimes when I got bored, they let me work a little bit–I helped the kitchens one day, helped with inventory–that was fun too” (Christophe, 37, translated from French).

The flexibility of the PRISM program–and the resulting freedom to maintain existing networks—was contrasted by some participants to previous experience with mental healthcare in a hospital setting:

“It’s not like a prison—I’ve never been to prison—but it’s… it’s isolation. It’s isolation, and… Yeah, that’s it. It’s like anything, you don’t want to feel too isolated because… because we need to have our means of expression, we need to have our freedom of movement, of spirit. But it’s a lot of isolation.” (Frédéric, 26, translated from French.)

#### A space for recovery

The second emergent theme was related to what many participants referred to as the “break” offered by PRISM, which enabled them to focus on their recovery. Our results suggest that through the simultaneous removal of some of the pressures of homelessness and the opportunity for flexible mental healthcare, participants were able to take some time for themselves and become engaged and involved in the development of their treatment plan.

Some participants arrived at the PRISM after years of chronic homelessness, others facing urgent housing needs for the first time. In either case, a majority of their resources had been directed towards meeting their basic needs of food and shelter. Frederic (26), who had spent the last few months using his old university locker as his only home base, emphasized how having his basic needs met was positive for him:

“And I didn’t necessarily have to rush because I already kind of had what I wanted. I’m fed, I’m housed. So it’s kind of like I was continuing my life in a way, I don’t have my full-full-full freedom, but… yeah.” (Frédéric, 26, translated from French.)

Chi (55) spoke similarly about the importance of having basic needs met:

“Security for my life because I don’t have to live outside. It’s very cold, the weather, and it’s very dangerous. And also here they have very good service, like you have breakfast, you have lunch, you have supper, and here even you have snack time, all kind of thing you need to eat you already have it. And you don’t have to spend your whole money.” (Chi, 55, original English quote.)

Many participants shared a general sense of relief and security upon arriving at PRISM:

“What I experienced a great deal of, what I find interesting is… I think this is an environment that is–for the 8 people who are at the PRISM, at least–it’s a safe environment. Especially with what I had been through when I got out of there [his last apartment].” (Pierre-André, 61, translated from French.)

The importance of this mental and physical “break” is best exemplified by participants’ views regarding their physical health: while many had noteworthy chronic physical ailments–diabetes, chronic pain, heart conditions, etc.–participants rarely identified these as priorities at intake, and fewer still had had much experience with physical health services. Nonetheless, there was a marked sense of relief concerning one’s physical health during exit interviews. Participants mentioned feeling generally better, attributing this to increase rest, regular meals and regular access to physical healthcare.

“There were things that I didn’t understand about my body. I had some anemia, my iron was low, I had just been operated on a month before coming here. Now, it’s going well, physically it’s going well, and my morale is good.” (Normand, 42, translated from French.)

For many, the challenges of meeting basic needs while homeless had been heavily compounded by their mental illness. This was illustrated well by Rémi (65) and the voices he was hearing: “Ah well, me, that’s how I’m built. I have a voice talking to me, and I have to follow. If I don’t follow, it’s: *Come on*, *come on*, *come on*, *come on*!*”* (Rémi, 65, translated from French). During this “break,” participants who had been facing chronic homelessness described an opportunity to get better psychologically:

“Well, for me, the main, big impact was to have… to be able to go from a state where–indeed, I look at that with some hindsight–I was truly manic, to being okay, you know. That’s what this gave–it was a moment of transition, you know, an important one.” (Pierre-André, 61, translated from French.)

Furthermore, some found the physical and mental respite to be conducive more generally to introspection, about themselves and their future: “But the impact, the impact… is to redefine, to define, what am I living presently, is it coherent for me? And for what I have to come, within society” (Baptiste, 46, translated from French). “It’s peaceful, this environment. We didn’t have the stress of people asking these questions every week. So it was really a moment of introspection for me” (Frédéric, 26, translated from French).

Similarly, Corey talks about the benefit of this zone of “safety” to delve into difficult issues in his life: “There’s like, many different aspects to it. But it gives you an opportunity to safely face things that are terrifying and know that you’ve got support in a ton of directions, so…” (Corey, 27, original English quote).

Furthermore, without the environmental pressures of survival, participants were granted the space necessary to consider the possibility of entering stable housing, which some had not seen as a viable option for a while: “It [my situation] got a lot better, because for me when I first got here I only thought about taking off, about leaving” (Rémi, 65, translated from French). Baptiste decided to settle into an apartment after more than 10 years of homelessness:

“I was trying to find shortcuts to say… to fight change a little bit. But in the end, it’s going well. I quickly resolved myself to say: “I think this will be the right thing to do,” so, to sign a lease and everything, and then we will see what we see for the future. And, I haven’t had an apartment in a long time, so… And, after years and years, well, you can be sure that the reflex, it’s a reflex of restraint rather than a reflex of openness, so I went towards the reflex of openness, for private, personal possibilities…” (Baptiste, 46, translated from French.)

André similarly commented on how this period allowed him to consider permanent housing:

“It gave me the visualization to know if I was ready to go into an apartment or not. To… with my customs, my customs in a little closed room, you know, to have a bed and to see if I was made to live in an apartment or if I was made to live on the street.” (André, 38, translated from French.)

Nevertheless, not all participants saw their time at PRISM as a secure respite. Some, particularly those who were younger than the mean, felt out of place in the PRISM setting. For example, Hugo (19) was especially disturbed by the “people who were fighting outside [adjoining shelter] and screaming at each other” (translated from French). He mainly focused his critique on one client with whom he had difficulty getting along: “But, it wasn’t because of the educators–or, the counsellors or whatever–it was more because of an old bastard who got on my nerves” (translated from French). He eventually preferred leaving the site while maintaining meetings with the staff: “I left the shelter [PRISM dorm] because I had trouble sleeping here. And… a few weeks ago I left to live with my mom (Hugo, 19, translated from French). Indeed, the social environment remained a source of discomfort for some participants. Pierre-André, for example, highlighted the difficulty of connecting with people whose symptoms of mental illness were more apparent:

“It’s that some of them are really, they’re… you talk to them and they are in their–we’re all in our story, that’s for sure. But at a certain point, you know…. It’s kind of quickly repetitive, the loop and spiral of what they are saying.” (Pierre-André, 61, translated from French.)

Finally, what had been hailed as a “break” by some was experienced as “boring” by others, especially those with limited shelter experience:

“Yeah, I appreciate the time I was able to take and stuff like that, but like… the other night I was, like, looking at this place and I was like, “Urgh. I just don’t want to go.” It’s fu…–it’s boring. It’s really boring. Like, the pillow is like a little sliver thing and you know, you’re in there with 8 guys and stuff like that… You really could go crazy. Then, at the same time… I am ambivalent, I guess, is a good word.” (Peter, 29, group 1, original English quote.)

#### Multimodal approach at the PRISM (compared to unimodal approach in the hospital)

The third emergent theme relates to the wide array of expertise found at PRISM, where participants were able to address a variety of issues in their lives; not only concerning their medication and housing, but also the general quality of their mental health and everyday lives. This multimodal aspect of PRISM was compared by some participants to their experience within the hospital system, where the scope of action may be limited to treating symptoms of mental illness.

Participants were overwhelmingly ambivalent about their past experiences with mental health services, be it with inpatient or outpatient care. For example, a recurring sentiment was that of ultimately having benefitted from a psychiatric stay, while highlighting the inhospitable nature of the experience, and its emphasis on medication: “I don’t know where they came up with the idea of giving these things [medication] to people and putting them in like, a square room, is going to somehow… It just seems so lazy” (Peter, 29, original English quote). “Do you need that kind of medication? […] Or you need some other helpful “beside,” *à côté*? You always need some *à côté*” (Chi, 55, original English and French quote).

Participants described how PRISM differed from previous experiences. Concerning medication changes, some felt appreciative that the on-site psychiatrist listened to and respected their concerns and requests about medication: “Me, I always asked [the doctor] that it be the most, well, as little as possible, you know, as little medication as possible. He respected my requests, and tried to diminish the dose as he could, little by little” (Pierre-André, 61, translated from French). “When I say that I’m not sleeping enough, or sleeping too much, [the doctor] is there to help me dose the medication better. It’s about teamwork, you know (Stéphane, 47, translated from French).

Some commented appreciatively on the talk therapy received through meetings with the psychiatrist, where they felt heard and understood without being judged: “It’s also a question of… listening, and really trying to understand and be empathetic, and trying to understand not just what [the person] says, but also how they feel, because our feelings also show a lot of information, you know” (Frédéric, 26, translated from French).

Participants appreciated the support received from the psychoeducator and the counsellor, as they were a near-constant presence that they could confide in if they felt the need.

“I didn’t—well, I didn’t feel judged, that’s important, I think. I think they had a good understanding of what was going on—they understood better than me what was going on, I think, and that’s really… that’s kind of what I was looking for by coming here.” (Christophe, 37, translated from French.)

Another aspect of this multimodal approach is that participants felt that they were not just passive recipients of care, instead being actively involved in their recovery. The program had a flexible structure allowing for such freedoms, while demanding more responsibility from clients than most psychiatric hospital stays: “The difference is that over there I was closely monitored, so for example for healthcare, the nurses or doctors came to me. […] Here, I have to go towards [them]” (Daniel, 20, translated from French).

Participants expressed a similar appreciation of PRISM’s patient-centred social services as they did its mental health services. As participants arrived at the PRISM with a variety of different needs and experiences, the program’s flexible structure enabled staff to respond uniquely to specific needs including applying for social assistance, obtaining identification, registering for an employment program, preparing to start a new job, applying for citizenship, dealing with legal situations, paying debts, dealing with substance abuse, etc. Participants contrasted this with their previous experiences looking for help: “When you ask for something, the response, it comes quickly, and… it’s done. Really no problem” (Pierre-André, 61, translated from French). “Often, every time I had a problem or that something was tough, well, often there was someone to help me with that, to… undo the knots, kind of. You know, it was something I couldn’t do on my own” (Christophe, 37, translated from French). “Yeah, it was good to have, just, I don’t know, just a break. A break from having to do everything on my own, you know?” (Peter, 29, original English quote).

As such, participants were empowered to make choices concerning their recovery, as they knew they would have the support to face administrative hurdles that had previously stood in their way: “The entire team is there if there is a problem […] Me, I’m the quarterback!” (Stéphane, 47, translated from French).

## Discussion

In this study, we set out to investigate the perceptions of PRISM users about the program in relation with their previous experiences with mental health resources, mostly inpatient psychiatric care. Three themes emerged from our analysis of the interviews: 1) the possibility to maintain one’s informal resource network, 2) a break from street life, and 3) multimodal care. From these results we will discuss three broader implications that were found to play a role in clients’ recovery. First, PRISM’s position within a shelter eased the transition from a homeless situation to housing. Second, the secure environment allowed participants to have the mental space to focus on housing. Third, the program’s individualized approach adapted to the unique needs and goals of participants. Overall, the experience may be more challenging for younger individuals without much experience with shelter life.

### Benefits of a shelter-based program

From its position within a shelter and permeability to the outside world, PRISM users could maintain an engagement with city life while receiving all their services in a central hub.

#### Engagement with urban life

Participants spoke of the ways receiving services in the shelter, rather than being confined in a hospital, allowed them to continue to be involved in the day-to-day activities that were part of their lives prior to being admitted to PRISM. For example, this could include hanging out at fast-food restaurants, hunting for returnable cans and bottles, going to the local library or spending time at a day program for people living in homelessness. Urban studies have been interested in the way homeless people make tactical use of spaces in the city in order to gather material or emotional sustenance, what Cloke has called the “homeless city” [27]. Our results suggest that recognizing and maintaining these informal networks ease the process of reintegration to housing and connection to the health care system.

Our analyses also highlight the importance of social relations both inside and outside PRISM as part of participants’ informal resource networks. Within PRISM, this involved collaboration between clients, exchange of goods, or volunteering in the shelter. Social networks outside PRISM were also maintained, be it with close friends and family, or acquaintances still living in homelessness. This can also include the day-to-day interactions that composed urban life. Ware et al. [28] have described how reciprocal interpersonal relationships generate a sense of connectedness which is primordial in social integration. The engagement with urban life through connection with people and activities may help clients to feel that they are “of the community” and not only “in the community’, to use the distinction made by the authors [28].

An important part of the therapeutic intervention with clients is identifying and guiding the participants towards resources, relationships and objectives that promote a path to recovery, and letting go of those that were destructive. For example, participants who use substances may be oriented toward a local Narcotics Anonymous group and accompanied there for the first meeting. Hence the PRISM staff can act as “brokers” between earlier and new forms of urban engagement.

#### Services available under one roof

Participants in our study highlighted how the availability of multiple types of expertise under one roof removed much of the red tape they had previously encountered, enabling them to seek out other resources with more assurance. Based on a qualitative study conducted with 40 homeless shelter residents in El Paso in the United Stated, Paat et al. [23] urged for the development of integrated services that take into account the broad array of needs in this population. According to the authors, a holistic approach that would include legal, relational, economic, physiological and psychiatric services, would increase user engagement. Meanwhile, existing services are often fragmented [29–31]: while the city of Montreal numbers over 93 public and non-profit resources dedicated to helping people living with housing instability, there is little formal and informal collaboration between these groups and the public health sector [32]. Recently, there have been a number of initiatives promoting integrated services [29–31], in which PRISM participates. Furthermore, many homeless individuals with mental illnesses may have conflicting, ambivalent perceptions of mental health and social services, and may refuse offers of help when such resources are made available [10, 33, 34]. In this context, rendering services as accessible as possible by making them available within the living environment may improve help-seeking in a homeless clientele.

### A breather to prepare for housing

In order to progress toward recovery, participants also described how it was necessary for them to have a respite from the hardship of life on the street and often a set of traumatic experiences. Participants not only had more time to address their physical and mental health, they were also given the space to approach the treatment and transition to housing at their own pace.

#### Time for mental and physical health care

Participants noted how their arrival at PRISM represented a notable change in pace, especially for those arriving directly from the street. Basic needs of food, shelter and security were ensured, freeing the considerable amount of physical and cognitive resources these needs had previously monopolized. Among other things, this respite offered by the PRISM environment allowed participants to take care of their physical and psychological health. Chronic physical health needs are known to be disproportionately high in homeless people with mental illness [3] and often only addressed in an emergency fashion, for example when one is faced with the life-threatening consequences of untreated diabetes [35]. Furthermore, many participants were ambivalent about past experiences with medication to treat mental disorders and appreciated the opportunity to take their time before accepting such treatment. Participants also valued talk therapy offered by the psychiatrist and counsellors, instead of mental healthcare geared exclusively toward medication. This diverges from previous research with this population, where participants expressed a “preference for practical help above ‘talking’ from their physicians and psychiatrists in outpatient therapy [21]. It is possible that by freeing participants from the constraints of meeting basic needs, the PRISM environment is more appropriate to talk therapy.

It does not come as a surprise that physical and mental health recovery is conditional upon meeting one’s basic needs. This highlights the importance of PRISM’s role in connecting or reconnecting participants to social follow-up services. By accompanying participants as they adapt to housing, these resources can assist with things like planning for paying rent or shopping for groceries, thus increasing the likelihood that basic needs will continue to be met.

#### Readiness for transition to housing

The PRISM environment granted the participant time and space to approach treatment milestones at their own pace: accepting medication, taking care of their physical health, taking time for psychotherapy, and transition to housing. Although a quick transition to independent housing is the end-goal of PRISM, the short shelter-based intermediary program offers a first step towards housing by providing security for someone who has been circling through the city’s streets, jails, emergency rooms and hospitals. Many participant testimonies highlight that reducing stress was conducive to introspection as one contemplated transitioning towards housing. This was true both for participants having spent many years on the street and for participants who found themselves in a homeless situation for the first time.

### Individualized care

The program’s flexibility allowed participants to feel that their individual needs were met and fostered a sense of agency. PRISM’s individualized approach to care may also allow it to address concerns from a minority of participants who felt ambiguous or negative about their stay.

#### Meeting the individual where they are

PRISM clients arrive at the program with widely different background, experiences and baseline levels of functioning. Seeing as each individual’s trajectory is unique, so must be the recovery plan. As such, our analyses overlap with recovery theory through the focus of *meeting people where they are* [36].

In a literature review concerning the perceptions of recovery held by people living with SMI, Drake and Whitley [37] highlight that recovery is a *process* rather than an *outcome*, and as such can involve small steps. Indeed, for someone who has been isolated and living outside, something as simple as cigarette exchanges can be the initial step towards social reintegration. PRISM’s flexibility in accompanying each individual in their own journey also allows it not to be rigid in its objectives. PRISM favor independent housing but will accompany people who are not ready and choose otherwise. Thiswas for example the case for Marc, who continued to visit acquaintances with whom he was using alcohol but who accepted to go to an inpatient rehabilitation facility three weeks after the start of the PRISM program.

#### Agency

Our participants highlighted in different ways the importance of playing a role in the decision-making process regarding treatment and housing, in comparison to the rather paternalistic approach some experienced at the hospital. Staff were able to meet individuals according to their level of autonomy. For example, concerning mental health services received on-site, a majority of participants appreciated adjustments made, with their input, to their medication. Participants identified themselves as key actors in their own treatment plan, which one participant referred to as “being the quarterback.” The journey toward autonomy also involves an increasing sense of agency and self-efficacy [37]. Shared decision-making has been identified as key to recovery and client-centred methods [38], as evidence suggests that such methods lead to a better therapeutic alliance and to better patient outcomes through, among others, better compliance [39], increased help-seeking behaviours and increased involvement towards treatment [40, 41]. The fact that the program is voluntary and very rarely involves visible forms of coercion may also reinforce this broad feeling of autonomy.

#### Necessity of increased accompaniment for some participants

The PRISM environment was experienced as challenging by a minority of participants. First, while group life was deemed by many as a core positive aspect of their stay at the PRISM, it led to significant obstacles for some. For example, one young participant deemed his interpersonal difficulties with another client unresolvable. Second, the PRISM environment was not seen as a secure respite by all. For younger participants with less experience within shelters, this environment could be jarring. This was especially true when other arrangements were possible, such as this same client whose mother allowed visits and finally a return to live with her, despite reservations expressed by the PRISM team. This was also the case for another participant, a foreign exchange student, who lost his room in a university residence following a psychotic episode. Finally, another frequent complaint was boredom, as the program does not present agendas filled with activities outside of meetings with the staff or psychiatrist and visits to exterior services or possible housing. Boredom is common in community-based treatment for mental illness, and may affect outcomes negatively [42]. As it stands, PRISM may be better suited for individuals who have experience in shelters and on the street. Participants could benefit from frequent check-ins to address these concerns. In order to mitigate those challenges, another initiative could be to add peer support, a tenet of recovery-focused mental healthcare associated with improved mental health outcomes [43, 44]. Such a peer support model could pair PRISM users with previous participants who are now stably housed. In our opinion, these peer-support individuals could be present either during outreach when an individual is ambivalent about entering the program, or in the first two weeks of admission at PRISM when clients are adapting to the new environment.

## Limitations

One limitation to this study is the context of data collection, which took place on-site at the PRISM, and could have created a potential response bias. To minimize these effects, special care was taken to highlight the completely elective and confidential nature of the interview, which was conducted by a research assistant who was not communicating with the treatment team about interviews during the study period. A second limitation is that participants varied immensely in their mental states, especially during the intake interview. In some cases, symptoms of mental illness could interfere with the intelligibility of interview content. Third, our research omits women’s perspectives, as the data collection was conducted at a PRISM inside a men’s shelter. Future research should address the needs of homeless women, for example by conducting a study in the PRISM located in a women’s shelter. The voices of gender diverse individuals were also not included and participants were not asked about their gender identity. Including the perspective of transgender and gender diverse individuals would be useful given the barriers they may face when navigating homelessness and institutional settings [45]. Meanwhile, the present study contributes to a better understanding of men’s mental health.

## Implications for policy and future research

Our study tends to show that the respite period in the shelter allowed participants to meet basic needs and create space for progress in terms of mental and physical health. The shelter environment may also represent an alternative to hospitalization even before patients are fully stabilized, given the intensity of PRISM services. By providing extended high-quality care in a low-cost shelter environment, PRISM may therefore offer a way out of the dilemma between quality versus efficiency that mental health services increasingly face, as evident by trends towards shortened psychiatric stays [46, 47]. It would be relevant to know if PRISM users manage to continue having their basic needs met when they are back in the community. Furthermore, the transition from the hospital to outpatient care is often a challenge [11]. The increased psychological and material readiness afforded to participants through their time at PRISM may ease such transitions. It would also be interesting to assess if the engagement with the mental health team at PRISM translates into improved continuity of care with the mental health community team or outpatient team after the PRISM program. To answer these questions, the data collection for a complementary study is presently underway, in which we endeavour to contact all participants and invite them to complete an interview 12 months after their discharge from PRISM to assess their return in the community. Furthermore, the PRISM model also offers the possibility of creating therapeutic relationships and finding the best living arrangement option for participants for whom housing is not an immediate goal, contributing to filling a gap in services for such individuals. In a complementary study, we will also analyze quantitative data from a larger pool of PRISM clients, based on questionnaires and provincial administrative databases, which may allow us to determine participant characteristics that most predict housing stability after a PRISM intervention as well as change in patterns of health services utilization.

## Conclusion

This study presents insights from PRISM clients concerning the program and their histories of homelessness. It reveals three characteristics that were deemed to be positive by the participants: 1) an approach that allows participants to continue to utilize the official and non-official resources gleaned thus far; 2) a context of respite from the taxing nature of life on the street, and 3) a multimodal mental health and social service approach that helped clients progress in their recovery. This allowed us to discuss the importance of a shelter-based program, of a space that allows individuals to heal and prepare for housing, as well as the importance of individual care that meets people where they are and accompany them toward their own goals. Younger participants without experience of shelter life appear to have faced a number of challenges in the program. Even though this study took place in a specific program in Quebec, it may shed light more broadly on how to best meet the needs of homeless person experiencing homelessness.

## Supporting information

S1 Annex Original language quotes. All quotes can be found in order of appearance in their original English or French.
(PDF)
