# Supplementary material for: Insights from homeless men about PRISM, an innovative shelter-based mental health service
Source: PLoS One. 2021 Apr 22;16(4):e0250341. doi: 10.1371/journal.pone.0250341 (PMC8062052; doi:10.1371/journal.pone.0250341)
Supplement: S1 Annex — All quotes can be found in order of appearance in their original English or French. (PDF) [file pone.0250341.s001.pdf]

Original Language Quotes

All participant quotes included in *Insights from homeless men about PRISM, an innovative shelter-based mental health service* are included here in their original language, either English or French. For ease of use, original French quotes are juxtaposed with their translation as it appears in the article.

Quote as it appears in the article	Original French quote
"It's like is I was playing Zelda or Link, right? So, I have a frame, you know, I can go click the frame and go for a walk […], I can go to the store, at the store there's a computer, I'll go sit in my corner, go to another corner, get down, go get my coffee, because I went - I went to the other store, I didn't get my coffee there, because I don't get a refill." (André, 38 translated from French)	*C'est comme si je jouerais mettons à Zelda ou à Link, ok? Fait'que, j'ai un tableau, tsé, j'peux aller peser su'l tableau pour marcher […], j'peux aller à la boutique, à la boutique y'a un ordinateur, j'va m'assir dans mon coin, j'vais à un autre coin, j'descends, j'va chercher mon café, parce que j'ai été - j'ai été à l'autre commerce, j'ai pas pris mon café là, parce que j'ai pas un remplissage.*
"It was so helpful, for real, I never drank so much juice in my life! I was there, I would take some juice, no one said anything. […] It might sound stupid, but they would accommodate me, like, I knew a few McDonalds that never kicked me out (Frédéric, 26, translated from French).	*P: Ça m'a tellement aidé, pour vrai, j'ai jamais bu autant de jus de ma vie! J'étais là, j'prenais du jus, personne disait rien […]. Ça peut paraître con, mais ils' m'accommodaient, genre, je sais que y'a des McDo qui me sortaient jamais.*
'We pick up bottles together, and… Well, yesterday we made five bucks each. Five bucks is five bucks" (Rémi, 65, translated from French).	*On ramasse les bouteilles ensemble, pis... Ben, hier on a fait 5 piasses chaque. C'est toujours 5 piasses.*
"[other participant], he's the one who gave me the shoes… and, euh… I gave him cigarettes often, you know… I had some money and I gave him cigarettes, so he gave me shoes and like… a shirt and everything. So we were kind of like friends." (Frédéric, 26, translated from French)	*C'est lui qui m'a donné les souliers… pi euh… j'lui donnais souvent des cigarettes tsé… j'avais d'l'argent puis j'lui donnais des cigarettes, fait'que y' m'a donné des souliers puis genre... un chandail puis toute. Fait que on était comme en amis là.*

“I had a negative experience [with another client] but it became positive. And that, I’m proud of that. We understood each other. And now, he’s leaving this morning, and I have a heavy heart.” (Marcel, 64, translated from French)	*J'ai eu une expérience négative, mais c'est devenu positif. Pis ça, j'suis ben fier de ça. On s'est compris. Pis, là il' quitte ce matin, pis j'ai le coeur lourd.*
“Breaking my isolation, that’s what I had to do. Breaking my isolation and making new friends, and… Since we’re in a group – there’s eight of us, right – well, we don’t have a choice but to coexist, you know, we have to talk and everything.” (Stéphane, 47, translated from French)	*Briser mon isolement, c'est ça qu'il fallait que j'fasse. Briser mon isolement pis me faire des nouveaux amis, puis... vu qu'on est en groupe - ben, on est 8, là - ben, on n'a pas le choix d'se côtoyer, tsé, 'faut qu'on s'parle pis tout ça.*
“I met new friends. Uhm... I like the food here, I like the food here, yeah, the food is good. Uhm... it's downtown, you know, it's near the metro, it's easy to get around from here. Yeah, there's... there's a lot of good things about it, yeah.” (Marc, 51, original English quote)	
“Well, now, I don’t want to leave as much, because I’m eating very well, here I can take the metro, to go to the post office [P.O. box] every day, as it is now I’m going three times a week” (Rémi, 65, translated from French).	*Ben, là j'ai moins envie de partir, parce que là j'mange très bien, ici j'peux faire du métro, pour aller au bureau de poste à tous les jours, comme c'est là j'y vais trois fois par semaine.*
“Sometimes when I got bored, they let me work a little bit – I helped the kitchens one day, helped with inventory – that was fun too” (Christophe, 37, translated from French).	*J'ai fait - des fois quand j'm'ennuyais, ils' m'ont laissé travailler un peu - j'ai aidé la cuisine une journée, j'ai aidé à faire l'inventaire - ça c'est l'fun aussi, là.*
“It’s not like a prison – I’ve never been to prison – but it’s… it’s isolation. It’s isolation, and… Yeah, that’s it. It’s like anything, you don’t want to feel too isolated because… because we need to	*C'est pas comme une prison - j'suis jamais allé en prison - mais c'est... c'est de l'isolation. C'est l'isolation, pis... Ouais, c'est ça. C'est comme toute autre chose, tu veux pas être trop isolé parce que... parce qu'on*

have our means of expression, we need to have our freedom of movement, of spirit. But it's a lot of isolation." (Frédéric, 26, translated from French)	*a besoin d'avoir nos moyens d'expression, on a besoin d'avoir notre liberté de mouvement, d'esprit. Mais c'est beaucoup d'isolation.*
"And I didn't necessarily have to rush because I already kind of had what I wanted. I'm fed, I'm housed. So it's kind of like I was continuing my life in a way, I don't have my full-full-full freedom, but… yeah." (Frédéric, 26, translated from French)	*Et pas nécessairement rusher parce que j'avais déjà un peu c'que je voulais. J'suis nourri, j'suis logé. Alors c'est comme si je continuais ma vie, mais dans le fond, j'ai pas toute, toute, toute ma liberté, mais… ouais.*
"Security for my life because I don't have to live outside. It's very cold, the weather, and it's very dangerous. And also here they have very good service, like you have breakfast, you have lunch, you have supper, and here even you have snack time, all kind of thing you need to eat you already have it. And you don't have to spend your whole money." (Chi, 55, original English quote)	
"What I experienced a great deal of, what I find interesting is… I think this is an environment that is – for the 8 people who are at the PRISM, at least – it's a safe environment. Especially with what I had been through when I got out of there [his last apartment]." (Pierre-André, 61, translated from French)	*C'que j'ai vécu beaucoup, c'que trouve intéressant c'est... j'trouve c'est un environnement - pour, en tout cas, les huit qui sont au PRISM - c'est un environnement sécure. Surtout avec c'que j'avais vécu en sortant de là-bas...*
"There were things that I didn't understand about my body. I had some anemia, my iron was low, I had just been operated on a month before coming here. Now, it's going well, physically it's going well, and my morale is good." (Normand, 42, translated from French)	*Il y a eu des affaires que je comprenais pas de mon corps. J'ai fait de l'anémie, mon fer était bas, je venais d'être opéré, un mois avant de rentrer ici. Là, ça va bien, physiquement ça va bien, moralement ça va bien.*

“Ah well, me, that’s how I’m built. I have a voice talking to me, and I have to follow. If I don’t follow, it’s: *Come on, come on, come on, come on!”* (Réal, 65, translated from French)	*Ah ben moi, j'suis amanché de même, moi. J'ai une voix qui me parle, pis qui faut que j'suive. Si je suis pas, c'est enweille, enweille, enweille, enweille.*
“Well, for me, the main, big impact was to have… to be able to go from a state where – indeed, I look at that with some hindsight – I was truly manic, to being okay, you know. That’s what this gave – it was a moment of transition, you know, an important one.” (Pierre-André, 61, translated from French)	*Ben, moi le gros gros impact c'est d'avoir - de pouvoir avoir passé d'un état où - effectivement, je regarde ça après recul - j'étais vraiment en manie, à être correct, tsé. C'est ça que ça m'a - ça a été un moment de transition, tsé, important.*
“But the impact, the impact… is to redefine, to define, what am I living presently, is it coherent for me? And for what I have to come, within society” (Baptiste, 46, translated from French).	*Mais, l'impact, l'impact... c'est de redéfinir, de définir, qu'est-ce que je vis présentement, est-ce que c'est cohérent pour moi? Et puis pour la suite de moi-même, dans la société.*
“It’s peaceful, this environment. We didn’t really have the stress of people asking these questions every week. So it was really a moment of introspection for me” (Frédéric, 26, translated from French).	*C'est paisible comme environnement. On n’avait pas vraiment eu de stress à personne qui demandait ces questions-là à chaque semaine. Alors c'était vraiment un moment d'introspection pour moi.*
“There's like, many different aspects to it. But it gives you an opportunity to safely face things that are terrifying and know that you've got support in a ton of directions, so...” (Corey, 27, original English quote).	
“It [my situation] got a lot better, because for me when I first got here I only thought about taking off, about leaving” (Rémi, 65, translated from French).	*Oui, elle s'est améliorée beaucoup, parce que moi quand j'suis rentré ici j'pensais rien'que de décoller, de partir.*
“I was trying to find shortcuts to say… to fight change a little bit. But in the end, it’s going well. I quickly resolved myself to say: “I think this will be the	*Donc, trouver des moyens raccourcis pour dire... Pour combattre un petit peu la nouveauté. Mais en fin de compte, ça va assez bien. J'me suis vite résolu de dire:*

right thing to do," so, to sign a lease and everything, and then we will see what we see for the future. And, I haven't had an apartment in a long time, so… And, after years and years, well, you can be sure that the reflex, it's a reflex of restraint rather than a reflex of openness, so I went towards the reflex of openness, for private, personal possibilities…" (Baptiste, 46, translated from French)	*"J'pense que ça va être la bonne chose à faire", donc, de signer un bail et ainsi de suite, et ensuite on verra ce qu'on verra pour la suite des choses. Et puis, ça faisait longtemps que j'ai pas d'appartement, donc... Puis, après des années, des années, ben, c'est sûr et certain que le réflexe, c'est un réflexe plus de retenu qu'un réflexe d'ouverture, donc j'ai été vers le réflexe avec plus d'ouverture, pour des possibilités privées, personnelles.*
"It gave me the visualization to know if I was ready to go into an apartment or not. To… with my customs, my customs in a little closed room, you know, to have a bed and to see if I was made to live in an apartment or if I was made to live on the street." (André, 38, translated from French)	*Ça m'a donné la visualisation de si j'étais prêt à aller en appartement ou pas. De… avec mes coutumes, mes coutumes dans une p'tite chambre fermée, tsé, d'avoir un lit pis d'voir si j'étais pour être en appartement ou si j'étais fait pour vivre dans la rue.*
"people who were fighting outside [adjoining shelter] and screaming at each other" (Hugo, 19, translated from French).	*Pis le monde qui se bataillait dehors ou qui se criait dessus, là.*
"But, it wasn't because of the educators – or, the counsellors or whatever – it was more because of an old bastard who got on my nerves" (Hugo, 19, translated from French).	*Mais, c'était pas à cause des éducateurs - euh, l'intervenant ou whatever - c'était plus à cause d'un vieux criss qui me tapait sur les nerfs.*
"I left the shelter [PRISM dorm] because I had trouble sleeping here. And… a few weeks ago I left to live with my mom (Hugo, 19, translated from French).	*J'suis parti du refuge parce que j'avais de la difficulté à dormir ici. Pis... v'la quelques semaines j'suis parti vivre avec ma mère.*
"It's that some of them are really, they're… you talk to them and they are in their – we're all in our story, that's for sure. But at a certain point, you know…. It's kind of quickly repetitive, the kind of loop and spiral of what they are saying." (Pierre-André, 61, translated from French)	*C'est parce que y'en a certains qui sont vraiment, sont... tu jases avec, pis 'sont dans leur - on est tous dans notre histoire, tsé ça c'est clair. Mais un moment donné, tsé c'est comme... c'est comme vite redondant, l'espèce de boucle pis spirale pis c'qu'ils' se disent, pis...*

"Yeah, I appreciate the time I was able to take and stuff like that, but like… the other night I was, like, looking at this place and I was like, "Urgh. I just don't want to go." It's fu… – it's boring. It's really boring. Like, the pillow is like a little sliver thing and you know, you're in there with 8 guys and stuff like that… You really could go crazy. Then, at the same time… I am ambivalent, I guess, is a good word." (Peter, 29, group 1, original English quote)	
"I don't know where they came up with the idea of giving these things [medication] to people and putting them in like, a square room, is going to somehow… It just seems so lazy" (Peter, 29, original English quote).	
"Do you need that kind of medication? […] Or you need some other helpful "beside," *à côté*? You always need some *à côté*" (Chi, 55, original English and French quote).	
"Me, I always asked [the doctor] that it be the most - well, as little as possible, you know, as little medication as possible. He respected my requests, and tried to diminish the dose as he could, little by little" (Pierre-André, 61, translated from French).	*Moi j'ai tout le temps demandé au [docteur] que ça soit le plus - en fait, le moins possible, tsé, le moins de médicamentation possible. Y'a respecté mes demandes, pis y'essayait de diminuer au fur et à mesure, le plus possible.*
"When I say that I'm not sleeping enough, or sleeping too much, [the doctor] is there to help me dose the medication better. It's about teamwork, you know (Stéphane, 47, translated from French).	*Quand j'dis que j'dors pas assez, ou j'dors trop, y'est là pour m'aider à mieux doser ma médication. C'est un travail d'équipe, tsé...*
"It's also a question of… listening, and really trying to understand and be empathetic, and trying to understand not just what the person says but also how they feel, because our	*C'est une question de… être à l'écoute puis de vraiment essayer de comprendre puis être empathique, pis essayer de comprendre, pas juste c'qu'à dit, mais comment qu'à se sent aussi parce que*

feelings also project a lot of information, you know.	*nos sentiments projettent beaucoup d'information aussi, tsé.*
"I didn't – well, I didn't feel judged, that's important, I think. I think they had a good understanding of what was going on – they understood better than me what was going on, I think, and that's really… that's kind of what I was looking for by coming here." (Christophe, 37, translated from French)	*J'me suis pas - ben, j'me suis pas senti jugé - ça, c'est important, j'pense. J'pense y'avait une bonne compréhension de ce qu'il' se passait - il' comprenait mieux qu'est-ce qu'il se passait que moi, j'pense, pis ça c'est vraiment - c'est un peu ça que j'venais chercher.*
"The difference is that over there… I was closely monitored, so for example for healthcare, the nurses or doctors came to me. […] Here, I have to go towards… [them]" (Daniel, 20, translated from French).	*La différence c'est que là-bas… j'étais suivi de près donc par exemple pour les soins de santé, les infirmiers ou les médecins venaient à moi… Alors qu'ici c'est moi qui dois aller vers… [eux].*
"When you ask for something, the response, it comes quickly, and… it's done. Really no problem" (Pierre-André, 61, translated from French).	*Quand y'a une demande, la réponse, elle vient rapidement, pis c'est… c'est rendu. Vraiment zéro problème.*
"Often, every time I had a problem or that something was tough, well, often there was someone to help me with that, to… undo the knots, kind of. You know, it was something I couldn't do on my own" (Christophe, 37, translated from French).	*Souvent à chaque fois que j'rencontrais un problème ou que y'avait quelque chose de difficile, ben, pas mal souvent y'a quelqu'un qui était là pour m'aider avec ça, un peu pour... défaire les noeuds, mettons. Tsé, c'est quelque chose que j'étais pas capable de faire tout seul…*
"Yeah, it was good to have, just, I don't know, just a break. A break from having to do everything on my own, you know?" (Peter, 29, original English quote).	
"The entire team is there if there is a problem […] Me, I'm the quarterback!" (Stéphane, 47, translated from French).	*Toute l'équipe est là pour un problème […] Moi j'suis quart-arrière!*
